# ACKR4 Recruits GRK3 Prior to β-Arrestins but Can Scavenge Chemokines in the Absence of β-Arrestins

**DOI:** 10.3389/fimmu.2020.00720

**Published:** 2020-04-22

**Authors:** Christoph Matti, Angela Salnikov, Marc Artinger, Gianluca D'Agostino, Ilona Kindinger, Mariagrazia Uguccioni, Marcus Thelen, Daniel F. Legler

**Affiliations:** ^1^Biotechnology Institute Thurgau (BITg) at the University of Konstanz, Kreuzlingen, Switzerland; ^2^Institute for Research in Biomedicine, Università della Svizzera Italiana, Bellinzona, Switzerland; ^3^Faculty of Biology, University of Konstanz, Konstanz, Germany; ^4^Theodor Kocher Institute, University of Bern, Bern, Switzerland

**Keywords:** atypical chemokine receptor, ACKR4, CCL19, CCL21, CCL25, β-arrestin, GRK3

## Abstract

Chemokines are essential for guiding cell migration. Atypical chemokine receptors (ACKRs) contribute to the cell migration process by binding, internalizing and degrading local chemokines, which enables the formation of confined gradients. ACKRs are heptahelical membrane spanning molecules structurally related to G-protein coupled receptors (GPCRs), but seem to be unable to signal through G-proteins upon ligand binding. ACKR4 internalizes the chemokines CCL19, CCL21, and CCL25 and is best known for shaping functional CCL21 gradients. Ligand binding to ACKR4 has been shown to recruit β-arrestins that has led to the assumption that chemokine scavenging relies on β-arrestin-mediated ACKR4 trafficking, a common internalization route taken by class A GPCRs. Here, we show that CCL19, CCL21, and CCL25 readily recruited β-arrestin1 and β-arrestin2 to human ACKR4, but found no evidence for β-arrestin-dependent or independent ACKR4-mediated activation of the kinases Erk1/2, Akt, or Src. However, we demonstrate that β-arrestins interacted with ACKR4 in the steady-state and contributed to the spontaneous trafficking of the receptor in the absence of chemokines. Deleting the C-terminus of ACKR4 not only interfered with the interaction of β-arrestins, but also with the uptake of fluorescently labeled cognate chemokines. We identify the GPCR kinase GRK3, and to a lesser extent GRK2, but not GRK4, GRK5, and GRK6, to be recruited to chemokine-stimulated ACKR4. We show that GRK3 recruitment proceded the recruitment of β-arrestins upon ACKR4 engagement and that GRK2/3 inhibition partially interfered with steady-state interaction and chemokine-driven recruitment of β-arrestins to ACKR4. Overexpressing β-arrestin2 accelerated the uptake of fluorescently labeled CCL19, indicating that β-arrestins contribute to the chemokine scavenging activity of ACKR4. By contrast, cells lacking β-arrestins were still capable to take up fluorescently labeled CCL19 demonstrating that β-arrestins are dispensable for chemokine scavenging by ACKR4.

## Introduction

Chemokines, a group of about 50 chemotactic cytokines, have fundamental roles in regulating immune responses, primarily by orchestrating leukocyte migration and controlling their localization (1, 2). The biological functions of chemokines are typically mediated by signaling through seven-transmembrane spanning, G-protein coupled receptors (GPCRs) (3, 4). The chemokines CCL19 and CCL21 are essential for guiding dendritic cells and subsets of T cells to lymph nodes by signaling through the cognate chemokine receptor CCR7 (5, 6) and thereby initiate adaptive immune responses. Notably, canonical CCR7 signaling by CCL19 and CCL21 is controlled by the G_i_ subfamily of G-proteins (7–9). In addition, chemokines bind to a small family of atypical chemokine receptors (ACKRs), which are structurally related to GPCRs but seem unable to elicit canonical, G-protein-dependent signal transduction pathways upon ligand binding (10, 11). However, ACKRs are emerging as crucial regulators for the availability of chemokines. Namely, ACKRs function as “decoy” or “scavenger” receptors that progressively internalize chemokines and sort them for lysosomal degradation to limit local and systemic chemokine concentrations (10, 12). The atypical chemokine receptor ACKR4, formerly also known as CCRL1 and CCX-CKR, is the scavenging receptor for CCL19, CCL21, and CCL25 (13–16). Notably, mice lacking ACKR4 systemically have a 5-fold increase in the level of CCL21 in the blood and a 2- to 3-fold increase in CCL19 and CCL21 in peripheral lymph nodes (17). Despite its expression on thymic epithelial cells where ACKR4 is supposed to scavenge the CCR7 and CCR9 ligands CCL19/CCL21 and CCL25, respectively, mice lacking ACKR4 seem to have a fairly normal thymic T cell lymphopoiesis (18). By contrast, ACKR4 expression by skin keratinocytes and a subset of dermal endothelial cells is critical for shaping functional CCL19/CCL21 gradients under steady-state and inflammatory conditions (17). These local CCL19/CCL21 gradients are essential for allowing dendritic cells to egress the skin and enter lymphatic vessels (19, 20). In addition, ACKR4 is present on lymphatic endothelial cells lining the ceiling of the subcapsular sinus, but not on those lining the floor, forming local CCL21 gradients in lymph nodes to guide dendritic cell homing in a CCR7-dependent manner (21). Consequently, the frequency of dendritic cells in the skin of ACKR4 deficient mice increases and dendritic cells fail to efficiently egress and migrate to draining lymph nodes (20, 21).

Although information on how ACKRs fulfill their scavenging function is limited, ACKR2-4 are known to spontaneously traffic between the plasma membrane and endosomes. Upon ligand binding, ACKRs internalize cognate chemokines and sort them for lysosomal degradation in a G-protein independent manner (10, 12, 16). The ability of ACKRs to scavenge chemokines has been linked to β-arrestins (22–26), which are universal intracellular adaptor proteins of GPCRs (27). In the case of classical GPCRs, β-arrestin recruitment depends on agonist-driven phosphorylation of serine/threonine residues situated at the receptor's C-terminus by GPCR kinases (GRKs) or other protein kinases and leads to clathrin-mediated receptor endocytosis (28). This general concept has recently been challenged, at least for ACKRs, as chemokine uptake by ACKR2 (29), ACKR3 (30, 31), and ACKR4 (16) was observed in cells lacking β-arrestins. By contrast, ligand-mediated β-arrestin recruitment to ACKR4 (26), ACKR3 (24), and ACKR2 (29), as well as subsequent β-arrestin-dependent activation of Erk1/2 through ACKR3 (24) was reported. These controversial data prompted us to in depth investigate early signal transduction pathways and chemokine scavenging activities of ACKR4.

In the present study, we provide evidence that ACKR4 neither interacts with nor activates heterotrimeric G-proteins. Chemokine binding to ACKR4 does also not activate canonical chemokine receptor kinases, such as Erk1/2, Akt, or Src. By contrast, chemokine triggering recruited β-arrestin1 and β-arrestin2 to ACKR4. Moreover, we identify GRK3, and to a lesser extent GRK2, as interaction partner of chemokine engaged ACKR4 and show that GRK3 recruitment precedes the recruitment of β-arrestins upon receptor triggering. We further demonstrate that the C-terminus of ACKR4 is critical for spontaneous receptor trafficking, β-arrestin recruitment and chemokine scavenging. Strikingly, overexpression of β-arrestins increased chemokine uptake by ACKR4, whereas in the absence of β-arrestins ACKR4 was still able to take up cognate chemokines although to a lesser extent, thus providing clear evidence that β-arrestins are dispensable for chemokine scavenging.

## Materials and Methods

### Bioinformatics

Phosphorylation site and kinase interaction site predictions were performed using the native human ACKR4 sequence (uniport: Q9NPB9) and the webservers NetPhos Server 2.0 (http://www.cbs.dtu.dk/services/NetPhos/) (32), NetphosK 1.0 (www.cbs.dtu.dk/services/NetPhosK) (33), and the ELM resource (34). Secondary structure predictions were made using NetSurfP-2.0 (35) and human uniprot sequences (GNAI: P63096, GNAO: P09471, GNAQ: P50148, GNAS2: P63092, GNA13: Q14344).

### Generation of Expression Plasmids

Reagents for molecular biology were purchased from Thermo Fisher Scientific and custom-designed primers from Microsynth. An overview of chemokine, receptor and β-arrestin constructs with corresponding primer sequences used for cloning are listed in Table 1. Briefly, pcDNA3 β-arrestin2i1-NLuc was generated by amplification of human β-arrestin2 and NLuc and subsequent ligation of the two PCR products over a common ClaI restriction site, followed by subcloning the DNA conjointly into the HindIII and XbaI sites of pcDNA3. Chemokines were amplified by PCR and further cloned into the XhoI and BsaI restriction sites of pET-His_6_-SUMO (41). SUMO-hCCL19-S6 was amplified by PCR and cloned into the XhoI and XbaI restriction sites of pET-His_6_-SUMO (41).

**Table 1:** General plasmids and primers.

**Construct**	**Template (if not synthesized); amplified insert in bold; [reference]**	**5′-forward primer**	**5′-reverse primer**	**Linker**
pcDNA3 β-arrestin2i1-Nluc	pcDNA3 **β-arrestin2i1**-Y2	GGTGGAAAGCTTATGGGGGAGAAACCCG	GCATCGATCCACCGCAGAGTTGATCATCATAGTC	^*^
	pAAVS1P-iCLHN **Nluc** (Addgene plasmid # 66579) (36)	CAATCGATCCACCGCTACCGCCACCGCCGGAACCGCCACCACCAGAACCGCCACCTCCGCCCGCCAGAATGCGTTC	GACCCAAGCTTGCCACCATGGTCTTCACACTCGAAGATTTCGTTGG	
pcDNA3 β-arrestin1A-Nluc	**β-arrestin1A** RC201279 (Origine)	GGTGGAAAGCTTATGGGCGACAAAGGGACCCG	GCATCGATCCACCTCTGTTGTTGAGCTGTGGAGAGCC	^*^
pEYFP β-arrestin2-EYFP	Published in (37)	-	-	
pcDNA3 hACKR4-EYFP	pcDNA3 CCR7-**EYFP** (9)	GGACTCGAGAGCGGAGGTGGCGGTTCTGGTGGTGGCGGTTCCGGCGGTGGCGGTAGCGTGAGCAAGGGCGAGGAG	GAATAGGGCCCTCTAGACTACTTGTACAGCTCGTCCATGC	^**^
pcDNA3 hACKR4t-EYFP	pcDNA3 **ACKR4**-EGFP (38)	GGAGACCCAAGCTTCATTACGATGGC	CTCTCGAGTCCACCAACGTACAAGATTGGGTTCAAACAAGAGTG	^**^
pcDNA3 hACKR4-mTq2	pcDNA3.1(-)Galphai1-**mTurquoise2** (39)	GCAGACTCGAGAGCGGAGGTGGCGGTTCTGGTGGTGGCGGTTCCGGCGGTGGCGGTAGCATGGTGAGCAAGGGCGAGG	GCAGGTCTAGATTACTTGTACAGCTCGTCCATGCCGAGAGTGATCCCGGCGGCG	^**^
pcDNA3 hACKR4-HA	pcDNA3 **ACKR4**-EGFP (38)	GGAGACCCAAGCTTCATTACGATGGC	CTACCTCGAGCCCCAATAGAGAAGGTAGAAGT	^***^
pcDNA3 hCCR7-EYFP	pcDNA3 **CCR7**-HA (40)	GACCCAAGCTTGGTACCGAGCTCGGATC	GTAGCTCGAGTCCACCGGAGAAGGTGGTGGTGGTCTCG	^**^
pcDNA3 hCCR7-HA	Published in (40)	-	-	^***^
pSUMO hCCL19	pCR3-**hCCL19**-Fc (40)	GGTGCTCGAGTTAACTGCTGCGGCGCTTC	GACTAGGTCTCCGGTGGGGGCACCAATGATGCTGAAGACTG	-
pSUMO hCCL21	Published in (41)			-
pSUMO hCCL25	**hCCL25** RC222128 (Origene)	GACTAGGTCTCCGGTGGGCAAGGTGTCTTTGAGGAC	GTGCTCGAGTTACAGTCCTGAATTAGCTGATATCAGGAGGG	-
pSUMO hCCL19-S6	p**SUMO hCCL19**	CCCTCTAGAAATAATTTTGTTTAACTTTAAGAAGGAGATATACATATGG	CAGGTGCTCGAGTTATTAGTTCAGCAGGCGCAGCAGCCAGCTCAGGCTATCGCCGCTGCCGCCGCCGCCGCTACTGCTGCGGCGCTTCATCTTGG	^****^

*Linker sequence used between protein and tag*.

* *GSI(GGGGS)_3_*.

** *GGLES(GGGGS)_3_*.

*** GARA.

**** *SGGGGS*.

***** *(GGGGS)_3_GGS*.

Nluc-GRK2 was generated by amplifying human GRK2 and Nluc separately, ligating the PCR products over a common ClaI restriction site and cloning it conjointly into the HindIII and XbaI sites of pcDNA3. The other GRK constructs were prepared by replacing GRK2 with GRK3, GRK4, GRK5, and GRK6 using HindIII and ClaI or ClaI and XbaI, as listed in Table 2.

**Table 2:** GRK related plasmids and primers.

**Construct**	**Template (if not synthesized); amplified insert in bold; [reference]**	**5′-forward primer**	**5′-reverse primer**	**Linker**
pRK5-hGRK4 missing XbaI site	pRK5-hGRK4 (addgene: #32690) (42)	GCTTTGCCATTAGATCTCGACAAGAACATACATAC	GTATGTA TGTTCTTGTCGAGATCTAATGGCAAAGC	
pRK5-hGRK4 missing HindIII and XbaI site	pRK5-hGRK4 missing XbaI site	GTGAAAGTGAGGAAGCCTTGCCATTAGATCTCG	CGAGATCTAATGGCAAGGCTTCCTCACTTTCAC	
pcDNA3 Nluc-GRK2	pWZL Neo Myr Flag **ADRBK1**(addgene: #20418) (43)	GTAGCGGTGGATCGATGGGGTCTTCAAAATCTAAACCAAAGGACC	GATAGGGCCCTCTAGATCAGAGGCCGTTGGCACTGCCGCGCTGGACCAGCGGCACCTTGCTCAGCTCCACCACGGGCGAG	^*****^
	pcDNA3 β-arrestin2i1-**Nluc**	GACCCAAGCTTGCCACCATGGTCTTCACACTCGAAGATTTCGTTGG	CAATCGATCCACCGCTACCGCCACCGCCGGAACCGCCACCACCAGAACCGCCACCTCCGCCCGCCAGAATGCGTTC	
pcDNA3 GRK2-NLuc	pcDNA3 Nluc-**GRK2**	GAGACCCAAGCTTCATTACGATGGCGGACCTGGAG	GCAGCATCGATCCACCGAGGCCGTTGGCACTGC	^*^
pcDNA3 GRK3-Nluc	pDNR-Dual **GRK3** (DNASU: HsCD00022400)	GAGACCCAAGCTTCATTACGATGGCGGACCTGGAGG	GCAGCATCGATCCACCGAGGCCGTTGCTGTTTCTGTG	^*^
pcDNA3 Nluc-Grk4	pRK5-**hGRK4** missing HindIII and XbaI site	GTGGCGGTAGCGGTGGATCGATGGAGCTCGAGAACATCGTGGCCAAC	GAATAGGGCCCTCTAGATTAGCATTGCTTGGGTTCCACTTCCTTCTC	^*****^
pcDNA3 GRK5-Nluc	pWZL Neo Myr Flag **GRK5** (addgene: #20495) (43)	GAGACCCAAGCTTCATTACGATGGAGCTGGAAAACATCGTG	GCAGCATCGATCCACCGCTGCTTCCGGTGGAGTTC	^*^
pcDNA3 GRK6-Nluc	Synthesized	CTATAGGGAGACCCAAGCTTATGGAGCTCGAGAACATCGTAGCG	CCTCCAATCGATCCACCCCGCCAACTGCTGGTGGGGGCCTCGGGCTG	^*^

*Linker sequence used between protein and tag*.

* *GSI(GGGGS)_3_*.

***** *(GGGGS)_3_GGS*.

Site directed mutations of putative ACKR4 phosphorylation sites are listed in Table 3. Multiple site directed PCR were performed in consecutive cloning rounds to get ACKR4_TT_, ACKR4_SSS_, and ACKR4_TTSSS_ mutants.

**Table 3:** Phosphorylation site mutations in ACKR4-EYFP.

**Mutation**	**Nucleotide mutation**	**5′-forward primer**	**5′-reverse primer**
ACKR4_Y68F_	TA1103TT	GGTTGTTGCTATCTATGCTTTCTACAAGAAGCAAAG	CTTTGCTTCTTGTAGAAAGCATAGATAGCAACAACC
ACKR4_Y79F_	TA1136TT	GACCGATGTCTTCATTTTGAACTTGGCTGTTG	CAACAGCCAAGTTCAAAATGAAGACATCGGTC
ACKR4_Y138F_	TA1313TT	CATCTCTATTGATAGATTCGTTGCTGTTACCAAGG	CCTTGGTAACAGCAACGAATCTATCAATAGAGATG
ACKR4_T142A_	A1325G	GATACGTTGCTGTTGCCAAGGTCCCATCTC	GAGATGGGACCTTGGCAACAGCAACGTATC
ACKR4_S146A_	TCT1337GCC	CTGTTACCAAGGTCCCAGCCCAATCTGGTGTTGG	CCAACACCAGATTGGGCTGGGACCTTGGTAACAG
ACKR4_S148A_	TCT1343GCC	CAAGGTCCCATCTCAAGCCGGTGTTGGTAAACCATG	CATGGTTTACCAACACCGGCTTGAGATGGGACCTTG
ACKR4_T226A_	ACT1577GCC	GCTACTTCATTACCGCTAGAGCCTTGATGAAGATGCCAAACATC	GATGTTTGGCATCTTCATCAAGGCTCTAGCGGTAATGAAGTAGC
ACKR4_S236A_	T1607G	CAAACATCAAGATCGCCAGACCATTGAAGG	CCTTCAATGGTCTGGCGATCTTGATGTTTG
ACKR4_S309A_	TCT1826GCC	CGTTTTTATGGGTGCCGCCTTCAAGAACTACG	CGTAGTTCTTGAAGGCGGCACCCATAAAAACG
ACKR4_S323A_	TCT1868GCC	GCTAAGAAGTACGGTGCCTGGAGAAGACAAAGACAATC	GATTGTCTTTGTCTTCTCCAGGCACCGTACTTCTTAGC
ACKR4_S330A_	T1889G	GAAGACAAAGACAAGCCGTTGAAGAATTCCC	GGGAATTCTTCAACGGCTTGTCTTTGTCTTC

To generate the BRET constructs for G proteins, all redundant HindIII, ClaI, BamHI, XhoI, or XbaI sites were removed by introducing silent mutations as listed in Table 4. Then, a BamHI site encoded in a SGGGGS linker was introduced (Table 5, Supplementary Figure 3). Further, the modified Gα-subunits were amplified with adjacent HindIII and XbaI sites and cloned into pcDNA3. PCR amplified Nluc was introduced into the BamHI sites (Table 5). An exception is Gα_q_, were the RLuc8 in Gα_q_-RLuc8 was replaced via BamHI cutting and insertion of Nluc. To generate pIRES Gβ-2A-cpV-Gγ2 Gα-Nluc, a redundant HindIII was removed and a new one added after the IRES sequence using site directed mutagenesis. Then the mutated IRES sequence was amplified using the forward primer hybridizing at a SalI site and the reverse primer with a HindIII site, hybridizing to the one introduced beforehand, followed by an XbaI site at its end. The PCR-product was ligated into the original IRES plasmid, removing the Gαi2-mTurquoise2 sequence, after digesting both with SalI and XbaI. Gα_i_-Nluc was cut out from pcDNA3 Gα_i_-Nluc utilizing HindIII and XbaI and ligated into the modified IRES vector.

**Table 4:** Templates for Gα and site directed mutations thereof.

**Gα variant**	**Mutation effect**	**5′-forward primer**	**5′-reverse primer**
Gα_i/o/q_-RLuc8	A kind gift from Nevin Lambert (44)		
Gα_s_	Synthesized		
Gα_13_	A kind gift from B.Moepps. (45)		
pIRES Gß-2A-cpV-Gy2 GNAi3-mTq2	addgene #69625 (39)		
Gαo	– BamHI site	CGCAAGAAGTGGATTCATTGCTTCGAGGAC	GTCCTCGAAGCAATGAATCCACTTCTTGCG
GαS	– BamHI site	CATTGTGAAGCAGATGAGAATCCTGCATGTTAATGG	CCATTAACATGCAGGATTCTCATCTGCTTCACAATG
	– BamHI site	GCCGCAAGTGGATACAGTGCTTCAACG	CGTTGAAGCACTGTATCCACTTGCGGC
	+ BamHI site	CCCCCCGTGGAGCTGTCAGGTGGCGGATCCCAGTTCAGAGTGG	CCACTCTGAACTGGGATCCGCCACCTGACAGCTCCACGGGGGG
Gα13	– BamHI site	CATATTCCCTGGTCAGGTGGCGGATCCGGAGACAACTC	GAGTTGTCTCCGGATCCGCCACCTGACCAGGGAATATG
	– HindIII site	CTCGAGAGAAGCTCCATATTCCCTGGG	CCCAGGGAATATGGAGCTTCTCTCGAG
	+ BamHI site	CTATTTCCTAGAATTTGAAGGCGATCCCCACTGCTTAAGAGAC	GTCTCTTAAGCAGTGGGGATCGCCTTCAAATTCTAGGAAATAG
pIRES	– HindIII site	AATGTCGTGAAGGAAGCAGTACCTCTGGTAGCTTCTTGAAGACAAACAAC	TTGTTTGTCTTCAAGAAGCTACCAGAGGTACTGCTTCCTTCACGACATTC
	+ HindIII site	GTTTTCCTTTGAAAAACACGATGATAATAAGCTTTGCACGTTGAGCGCCGAAGACAAGGCGG	CCGCCTTGTCTTCGGCGCTCAACGTGCAAAGCTTATTATCATCGTGTTTTTCAAAGGAAAAC

**Table 5:** Templates for Gα and Nluc amplification.

**Affected protein**	**Amplification of**	**5′-forward primer**	**5′-reverse primer**
NLuc	BamHI-Nluc-BamHI	TCAGGTGGCGGATCCATGGTCTTCACACTCGAAGATTTCGTTG	GATGCCGGATCCTCCACCGCCAGAGCCCGCCAGAATGCGTTCGCAC
Gα_o_	HindIII-G**α**o(1)-BamHI	GAGACCCAAGCTTCAGCCACCATGGGATGTACTCTGAGCGCAGAGGAG	GGATCCGCCACCTGACAAAGTGTCCATGGCCCGGACGATGGCTGCCAGGGAC
	BamHI-G**α** o(2)-XbaI	TCTGGCGGTGGAGGATCCGGCATCGAATATGGTGATAAGGAGAGAAAG	CAGGGCCCTCTAGATCAGTACAAGCCGCCGCCCCGGAG
Gα_i_	HindIII-G**α**i(1)-BamHI	GGATCCGCCACCTGACAACCTCCCCATAGCCCTAATGATAGCAATAATTGACTG	GAGACCCAAGCTTCAGCCACCATGGGCTGCACGCTGAGC
	BamHI-G**α**i(2)-XbaI	TCTGGCGGTGGAGGATCCAAGATAGACTTTGGTGACTCAGCCCG	GTATGCCTCTAGATCAAAAGAGACCACAATCTTTTAGATTA
Gα_13_	HindIII-Gα_13_-XbaI	GACCCAAGCTTATGGCGGACTTCCTGCCGTC	CAGGGCCCTCTAGATTACTGTAGCATAAGCTGCTTGAGGTTGTC
Gα_S_	HindIII-Gα_S_-XbaI	GACCCAAGCTTATGGGCTGCCTC	CAGGGCCCTCTAGATTAGAGCAGCTCGTACTGACGAAGGTG
IRES sequence	amplification	GTTCGAAGTCGACAGATCTC	CATCGCTCTAGACGTACTAGCAAGCTTATTATCATCGTGTTTTTCAAAGG

### Chemokine Production

Recombinant human chemokines fused to a His_6_-SUMO-tag were purified from BL21 (DE3) *E. coli* and refolded by infinite dilution at pH 8.5. The His_6_-SUMO-tag was cleaved off by incubation with the Ulp-1 protease for 1–5 h and removed (41, 46, 47). Chemokines were purified by RP-HPLC on C18 columns.

To generate fluorescently tagged CCL19^Dy649P1^, human CCL19 fused to a His_6_-SUMO-tag and a SGGGGS-S6-tag was expressed and purified as described above. CoA-conjugated (C3144-25MG, Sigma) Dy649P1 (Dy649P1-03, Dyomics GmbH) was prepared as described (46). Fluorescently labeled CCL19^Dy649P1^ was generated by labeling purified CCL19-S6 with CoA-Dy649P1 at 37°C for 2 h using the phosphopantetheinyl transferase Sfp (P9302S, New England Biolabs) as previously described (46). Excess of substrate was removed from fluorescently labeled chemokine by reverse phase HPLC.

### Cell Culture and Transfection

HeLa cells were cultured in DMEM (P04-04510, Pan Biotech), containing 1% penicillin/streptomycin (Pan Biotech), 10% FBS (Thermo Fisher Scientific). Cells were transfected at least 30 h prior to the experiments using the 100 μl Neon® Transfection System (Thermo Fisher Scientific) according to the manufacturer's protocol, transfecting 5 × 10^5^ cells with 10 μg total plasmid DNA. For BRET recruitment experiments, the DNA ratio of fluorophore to luciferase construct was 3:1, for Gα_i_ activation experiments, the ratio of pcDNA3 receptor-HA to pIRES Gα-Nluc Gβγ-cpVenus construct was 1:3.

### Chemokine Mediated Erk1/2, Akt, and Src Activation

HeLa cells were transfected either with pcDNA3 ACKR4-HA, pcDNA3 CCR7-HA or empty pcDNA3. After 36 h, cells were starved for 2 h with medium containing 0.5% serum before they were stimulated with 1 μg/ml (114 nM) human CCL19. Cells were lysed using NP-40 lysis buffer as described (9). Samples were separated by SDS-PAGE and phosphorylated (p) and total (t) amounts of signaling proteins detected by Western blotting using the following antibodies purchased from Cell Signaling Technology: tErk (#9102) pErk (#4370), tSrc (#2109), pSrc (#6943), tAkt (#9272), pAkt (#9271).

### BRET Measurements

Transfected HeLa cells were grown in 6 well plates, washed with PBS, and detached using PBS based Gibco™ cell dissociation buffer (#13151014, Thermo Fisher Scientific) for a minimum of 3 min. Cells were collected in twice the volume of dissociation buffer with DMEM containing 10% FCS before being centrifuged for 2 min at 200 g. Cells were washed and resuspended in PBS containing 5% (w/v) glucose (PBS-G). Aliquots of around 8 × 10^4^ cells in 40 μl were inoculated in white 96-flat-bottom half-well plates in the presence of 5 μM luciferase substrate coelenterazine H (#C-7004, Biosynth) and stimulated with various concentrations of chemokines. Ratiometric BRET measurements were performed using a Tecan Spark™ 10M multimode microplate reader, measuring luciferase bioluminescence (384–440 nm, 350 ms integration time) and EYFP fluorescence (505–590 nm, 350 ms integration time) to calculate the BRET ratio between both signals (48). For short term observations (−1 to 3 min), the integration time of both signals was decreased to 250 ms and an injector used for chemokine addition. To calculate NetBRET, BRET ratio of control wells containing luciferase and HA-tagged receptor instead of EYFP-tagged receptor was subtracted from the sample BRET ratio. For Gα_i_ activation, the control wells contained cells transfected with pIRES Gα-Nluc Gβγ-cpVenus alone. Area under the curve analysis (AUC) was performed using the measurements before stimulation as baseline and integrating the peak starting from 0 min until the end of measurement. For data representation of GRK and G protein activation, baseline reduction was performed using the measurements before addition of ligands, which is referred to as “corrected NetBRET.”

### Chemokine Uptake Assay

Transfected HeLa cells were seeded at 4.5 × 10^4^ cells per well in 24 well plates. Cells were washed with PBS and incubated for at least 10 min in 200 μl 50 mM HEPES-buffered, high glucose DMEM without phenol red (#21063045 Thermo Fisher Scientific) at 37 or 8°C, respectively. Fifty microliter of chemokine solution was added to the cells for indicated times. At *t* = 0 min, all cells were washed twice with PBS; acidic wash (100 mM NaCl, 50 mM glycine HCL, pH 3.0) was applied to the designated wells for about 45 s, followed by two PBS washes. Cells were detached by incubation with PBS based Gibco™ cell dissociation buffer and subsequently measured on a BD LSR II flow cytometer and FACSDiva™ software (BD Biosystems). Data were analyzed using the FlowJo™ 10.7 software.

### ACKR4 Receptor Staining and Chemokine Binding

Transfected HeLa cells were seeded at 2.5 × 10^5^ cells per well in 6 well plates. About 24–36 h post-transfection, cells were washed with FACS buffer (145 mM NaCl, 5 mM KCl, 1 mM MgCl_2_, 1 mM CaCl_2_, 1 mM sodium phosphate, 5 mM HEPES, pH 7.5) and detached using Gibco Cell Dissociation Buffer (ThermoFisher). Cells were incubated with α-hACKR4 primary antibody (clone 13E11; #362102 Biolegend, dilution 1:750) at 8°C for 40 min followed by intense washing and incubation with goat α-mouse IgG coupled to Alexa647 (#A-21235 ThermoFisher, dilution 1:1000) for additional 20 min. To determine chemokine binding capacities to different ACKR4-EYFP mutants, transfected cells were incubated with 25 nM site specific labeled human CCL19 (CCL19^Dy649P1^) at 8°C for 30 min. After washing, cells were analyzed by flow cytometry on a LSR II (BD Biosciences). Flow cytometry data were analyzed using FlowJo V10 (BD Biosciences). Medians of chemokine or antibody fluorescence of EYFP^+^ cells were related to the median of EYFP to consider transfection efficiency.

### Confocal Fluorescence Microscopy

Transfected HeLa were seeded in 6 well plates containing 18 mm 1.5H glass slides (#0117580 Marienfeld-Superior). After 36 h, cells were fixed using 4%formaldehyde and 1%glutaraldehyde and subsequently stained with phallodin-Alexa647 and mounted with DAPI Fluoromount-G (#0100-20, SouthernBiotech). A Leica TCS SP5 II confocal microscope with a 63x oil-immersion objective was used. Acquired images were processed using Fiji (49) and ImageJ2 (50). For deconvolution of 3D stacks, SVI Huygens Essential version 16.10.0p3 was used.

### Data Analysis

**Figure 1:**
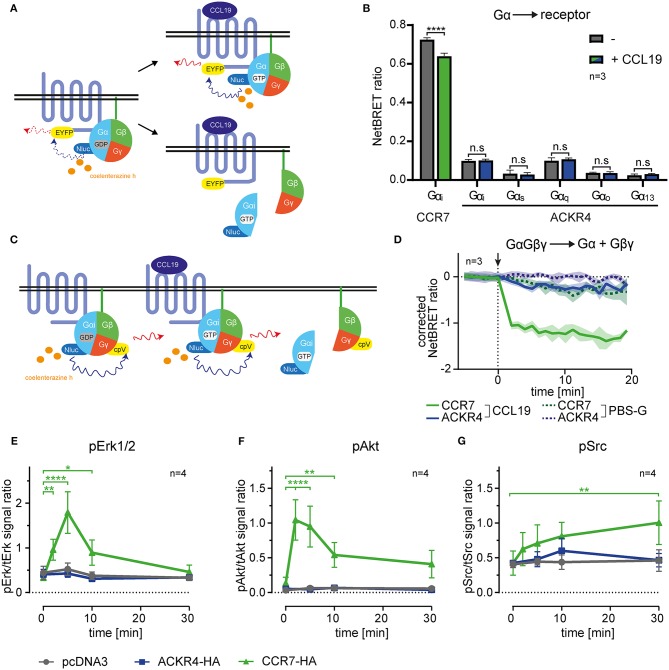
ACKR4 does not interact with or activate G-proteins. **(A)** Schematic representation of BRET-based G-protein interaction, recruitment and dissociation assays. **(B)** HeLa cells were transiently transfected with either human CCR7-EYFP or human ACKR4-EYFP conjointly with Nluc fused to either Gα_i_, Gα_q_, Gα_s_, Gα_o_, or Gα_13_. NetBRET was determined before and after stimulating cells with 1 μg/ml (114 nM) human CCL19. *n* = 3. **(C)** Schematic representation of BRET-based G-protein activation assays. **(D)** HeLa cells expressing either human CCR7 or human ACKR4 together with an IRES vector coding for Gα_i_-Nluc and Gβγ-cpVenus were stimulated at *t* = 0 (indicated by an arrowhead) with 1 μg/ml CCL19 (solid lines) or PBS-G as control (dashed lines). G-protein activation was determined by measuring chemokine-mediated dissociation of the Gβγ- from the Gα-subunit manifested by a decrease in NetBRET. Mean values and SEM of 3 independent experiments are shown. **(E–G)** Mock transfected (pcDNA3) and ACKR4-HA or CCR7-HA transfected HeLa cells were stimulated with 1 μg/ml CCL19 and total vs. phospho-Erk1/2 **(E)**, total vs. phospho-Akt **(F)**, and total vs. phospho-Src **(G)** was determined by Western blot densitometric analysis. Mean values and SEM of 4 independent experiments are shown.

**Figure 2:**
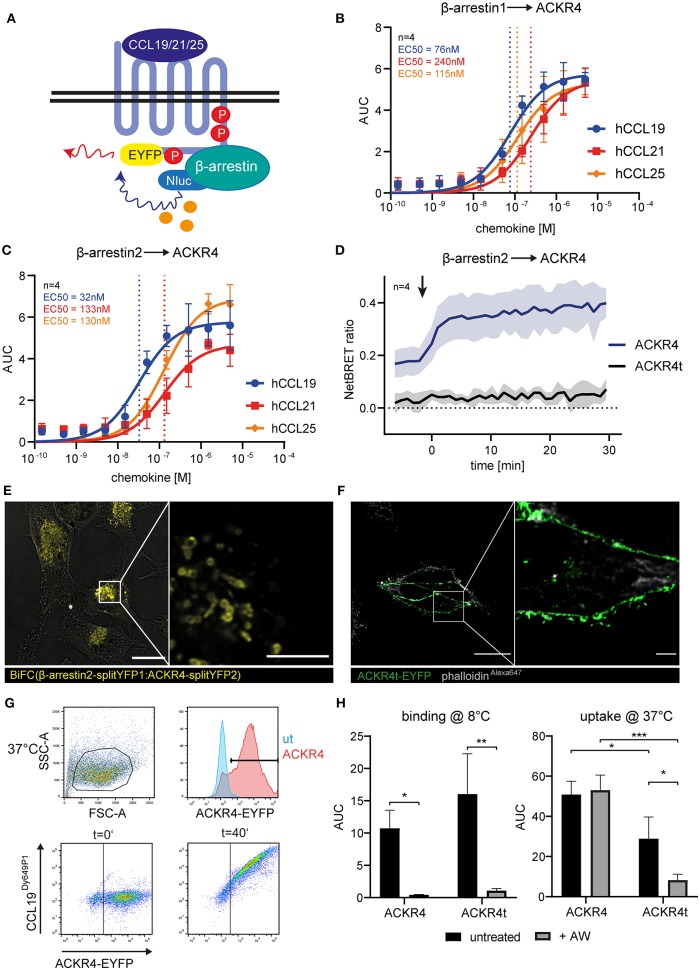
The C-terminus of ACKR4 is critical for β-arrestin recruitment and chemokine uptake. **(A)** Schematic representation of BRET-based β-arrestin recruitment assay. HeLa cells co-transfected with ACKR4-EYFP and β-arrestin1-Nluc **(B)** or β-arresin2-Nluc **(C)** were stimulated with graded concentrations of either human CCL19, CCL21, or CCL25 and β-arrestin recruitment to ACKR4 determined by BRET. *n* = 4. **(D)** HeLa cells co-transfected with β-arrestin2-Nluc together with ACKR4-EYFP or tailless ACKR4t-EYFP and stimulated at *t* = 0 min (indicated by an arrowhead) with 1.5 μM CCL19 and mean NetBRET and SEM derived from four individual experiments are depicted over time before and after chemokine addition. **(E)** HeLa cells were co-transfected with ACKR4-splitYFP2 and β-arrestin2-splitYFP1. BiFC was visualized by confocal microscopy under steady-state conditions. Scale bar = 25 μm or 3 μm for deconvoluted, zoomed image. **(F)** HeLa cells were transfected with ACKR4t-EYFP and its subcellular localization determined by confocal microscopy. A representative deconvoluted image is shown. Scale bar = 25 μm or 2.5 μm for zoomed image. **(G)** HeLa cells (ut) or HeLa cells expressing ACKR4-EYFP (ACKR4) were incubated at 37°C with 10 nM fluorescently labeled CCL19^Dy649P1^ for various time points. Receptor expression and chemokine uptake was determined by flow cytometry. **(H)** HeLa cells expressing ACKR4-EYFP or ACKR4t-EYFP were incubated with 10 nM fluorescently labeled CCL19^Dy649P1^ at either 8°C to determine chemokine binding, or at 37°C to determine chemokine uptake by flow cytometry for up 40 min. Where indicated, cells were shortly exposed to an acidic wash to remove surface bound chemokine. Mean values and SD of three independent experiments are shown.

Data analysis and presentation was performed using GraphPad Prism V.7 and V8. For statistics with one variable, RM one-way ANOVA or mixed-effects analysis, both with Dunnett's multiple comparisons test with a single pooled variance was performed (**Figure 4**). For statistics of Western blot ratio, mixed-effect model with Tukey's multiple comparisons test with a single pooled variance was performed (Figure 1). For experiments using two variables, ordinary two-way ANOVA with Tukey's multiple comparisons test, with individual variances computed for each comparison was performed (Figures 2, **5**). EC50 values were calculated fitting a three parameter [agonist] vs. response curve. ^*^p < 0.05, ^**^p < 0.005, ^***^p < 0.0005, ^****^p < 0.0001.

## Results

### ACKR4 Does Not Elicit Canonical Chemokine-Mediated Signal Transduction Pathways

ACKRs, including ACKR4, were reported not to signal through heterotrimeric G_i_-proteins manifested by the failure to induce cell migration or calcium mobilization (16, 26). However, it has been speculated that the G_i_-protein might associate with ACKR4, hence sterically block G_s_ activation unless it dissociates from the receptor (26). To address this possibility, we established bioluminescence resonance energy transfer (BRET)-based assays to measure G-protein activation and interaction with the receptor. We engineered human Gα-proteins (Gα_i_, Gα_q_, Gα_s_, Gα_o_, or Gα_13_) where we introduced the Nano luciferase (Nluc) as luminescence donor into the unstructured region after the second helix (51). As luminescence-acceptor, we used human ACKR4, or human CCR7, fused to EYFP to measure steady-state association, chemokine-driven recruitment, as well as activation-dependent dissociation of the heterotrimeric G-protein from the receptor (Figure 1A). Co-expressing Gα-Nluc variants together with ACKR4-EYFP in HeLa cells revealed that none of these tested Gα-proteins associated with ACKR4 under steady-state conditions (Figure 1B). Stimulating cells with 1 μg/ml of human chemokine CCL19, known to elicit strong CCR7 responses, neither recruited one of the Gα-proteins to ACKR4, nor resulted in the dissociation of one of the G-proteins from the receptor (Figure 1B). By contrast, Gα_i_ was found to pre-associate with the canonical chemokine receptor CCR7-EYFP and dissociated from the receptor upon CCL19 stimulation (Figure 1B), which is in line with the pre-association of the G_s_-protein with the adrenergic receptor and its subsequent ligand-induced dissociation (51). Next, we used Gα_i_-Nluc and Gβγ fused to cpVenus as luminescence-acceptor to monitor activation-induced dissociation of Gβγ from the Gα_i_-subunit (Figure 1C). Whereas, CCL19 stimulation led to the dissociation of the Gα_i_ from the Gβγ-subunit upon CCR7 triggering, this was not observed for ACKR4 (Figure 1D). Consistent with these findings, CCL19 triggering of CCR7, but not of ACKR4, lead to the phosphorylation and activation of the MAP kinase Erk1/2 (Figure 1E, Supplementary Figure 1) and protein kinase B/Akt (Figure 1F, Supplementary Figure 1) through the canonical G_i_-signaling pathway (7–9). Moreover, CCL19 stimulation of CCR7 caused the phosphorylation of the kinase Src (Figure 1G, Supplementary Figure 1), which occurs through G-protein-independent signaling (9). Again, no CCL19-mediated Src phosphorylation was observed upon ACKR4 triggering (Figure 1G, Supplementary Figure 1). These data provide comprehensive evidence that ACKR4 does neither associate with and activate G-proteins, nor elicits canonical chemokine receptor signaling pathways involving Erk1/2, Akt or Src kinases.

### The C-Terminus of ACKR4 Controls Interaction and Recruitment of β-Arrestins and Is Essential for Chemokine Uptake

As the role of β-arrestins in chemokine scavenging by ACKR4 is debated (16, 26), we determined β-arrestin1 and β-arrestin2 recruitment to ACKR4 by BRET (Figure 2A). We co-expressed EYFP-tagged human ACKR4 together with either Nluc-tagged human β-arrestin1 or β-arrestin2 in Hela cells and stimulated the cells with graded concentrations of the human ACKR4 ligands CCL19, CCL21, and CCL25. All three chemokines recruited β-arrestin1 (Figure 2B) and β-arrestin2 (Figure 2C) to ACKR4. CCL19 was the most potent agonist in recruiting β-arrestin1 (EC50~76 nM) and β-arrestin2 (EC50~32 nM). EC50 values for CCL21 were ~240 nM (β-arrestin1) and ~133 nM (β-arrestin2), those for CCL25 ~115 nM and ~130 nM, respectively (Figures 2B,C). As β-arrestin recruitment to a GPCR is controlled by phosphorylation of serine/threonine residues located at the receptor's C-terminus, we generated a tailless human ACKR4 variant by truncating the receptor directly after the conserved NPxxY motif (ACKR4_1−304_; termed ACKR4t). As expected, ACKR4t failed to recruit β-arrestin1 or β-arrestin2 upon chemokine stimulation (Figure 2D, Supplementary Figure 2). Notably, ACKR4t already showed a markedly reduced steady-state interaction with β-arrestins before the chemokine was added compared to wild-type ACKR4 (Figure 2D). To confirm and visualize β-arrestin interaction with ACKR4 under steady-state conditions, we exploited a split-YFP based biomolecular fluorescence complementation (BiFC) assay (9, 48, 52). We found that BiFC between ACKR4-splitYFP2 and β-arrestin2-splitYFP1 was predominantly found in vesicular structures (Figure 2E), suggesting that β-arrestins might contribute to the steady-state trafficking of ACKR4. Consistent with this hypothesis, ACKR4t (fused to EYFP) was predominantly expressed at the plasma membrane (Figure 2F). To assess chemokine scavenging, we incubated HeLa cells expressing either ACKR4-EYFP or ACKR4t-EYFP with fluorescently labeled CCL19 (CCL19^Dy649P1^) at either 8°C (to measure chemokine binding) or 37°C (to determine chemokine uptake; Figure 2G). At 8°C, both ACKR4 variants bound CCL19^Dy649P1^ with ACKR4t being slightly, but not significantly, more efficient (Figure 2H). Surface bound CCL19^Dy649P1^ was effectively removed by a short acidic wash (Figure 2H). Incubating ACKR4-EYFP expressing cells at 37°C resulted in a marked uptake of CCL19^Dy649P1^ which resisted the acidic wash, indicating that the chemokine was indeed rapidly internalized (Figure 2H). By contrast, using the same conditions, CCL19^Dy649P1^ bound to ACKR4t-EYFP, but was efficiently removed by an acidic wash (Figure 2H), revealing that ACKR4 lacking its C-terminus fail to efficiently take up CCL19.

### Chemokine Triggering Recruits GRK3, and to a Lesser Extent GRK2, to ACKR4

**Figure 3:**
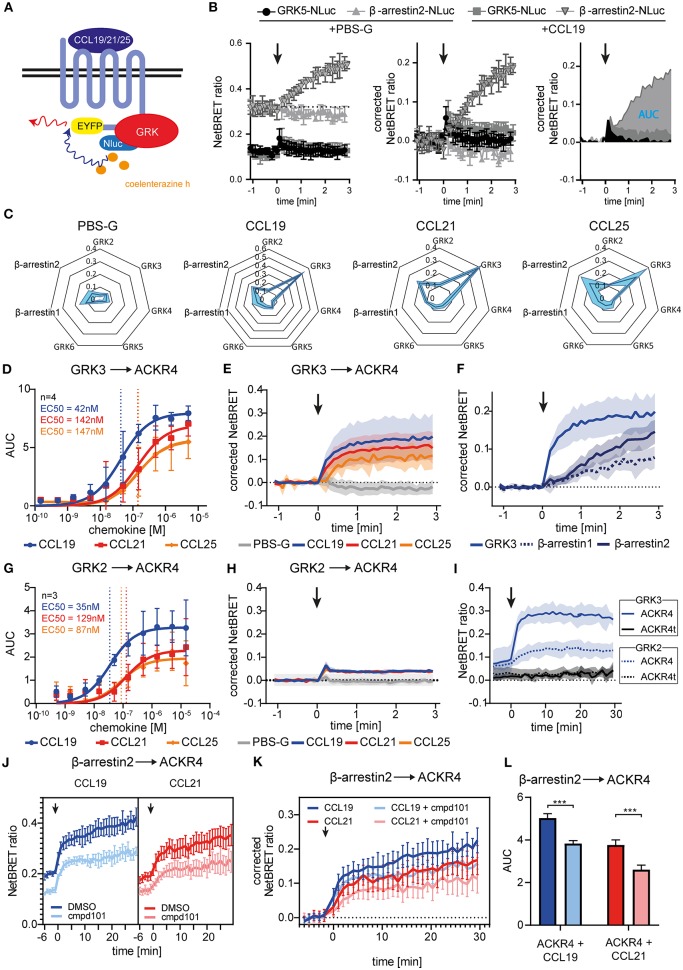
Chemokine stimulation selectively recruits GRK3 and GRK2 to ACKR4. **(A)** Scheme of chemokine-mediated GRK recruitment determined by BRET. **(B)** HeLa cells were co-transfected with ACKR4-EYFP and GRK5-Nluc or β-arrestin2-Nluc and stimulated with CCL19. Chemokine-mediated GRK5 or β-arresin2 recruitment was determined by NetBRET (left). NetBRET values before chemokine stimulation (baseline) was subtracted for corrected NetBRET values (middle). Corrected NetBRET values over time after chemokine addition was integrated and depicted as area under the curve (AUC) values (right). Chemokine addition is indicated by an arrowhead. Data from one representative experiment out of three experiments is shown. **(C)** Spider diagram of PBS or chemokine-mediated GRK2, GRK3, GRK4, GRK5, GRK6, β-arrestin1, and β-arrestin2 recruitment measured as AUC over 3 min. Chemokines were used at a concentration representing about 3-times the EC50 value of β-arrestin2 recruitment, namely 114 nM for CCL19, 408 nM for CCL21, and 352 nM for CCL25. *n* = 3. **(D)** Dose-response curve of GRK3-Nluc recruitment to ACKR4-EYFP upon ligand stimulation. *n* = 4. **(E)** Time-resolved GRK3-Nluc recruitment to ACKR4-EYFP upon 114 nM chemokine stimulation (indicated by an arrowhead). *n* = 3. **(F)** Time-resolved recruitment of GRK3-Nluc from **(E)**, β-arrestin1-Nluc, or β-arrestin2-Nluc to ACKR4-EYFP upon stimulation (indicated by an arrowhead) with 114 nM CCL19. *n* = 3. **(G)** Dose-response curve of GRK2-Nluc recruitment to ACKR4-EYFP upon ligand stimulation. *n* = 3. **(H)** Time-resolved GRK2-Nluc recruitment to ACKR4-EYFP upon 114 nM chemokine stimulation (arrowhead). *n* = 3. **(I)** GRK2 (dashed lines) and GRK3 (solid lines) recruitment to ACKR4-EYFP or ACKR4t-EYFP upon 114 nM CCL19 stimulation (arrowhead) over 30 min. *n* = 3. **(J–L)** HeLa cells transfected with ACKR4-EYFP and β-arrestin2-Nluc were pretreated for 2 h with 30 μM of the GRK2/3 inhibitor cmpd101 or solvent (DMSO) and subsequently stimulated with either 114 nM CCL19 or 408 nM CCL21 (indicated by an arrowhead). NetBRET **(J)** corrected NetBRET **(K)** or AUC **(L)** are depicted. *n* = 3.

To identify which GRK promotes putative receptor phosphorylation and subsequent β-arrestin recruitment we established BRET assays to measure recruitment of individual GRKs to engaged ACKR4 (Figure 3A). Therefore, we fused Nluc to all ubiquitously expressed human GRKs and co-expressed them individually with ACKR4-EYFP in HeLa cells. As internal control we also determined β-arrestin2-Nluc recruitment to ACKR4-EYFP. Cells were stimulated with the three ACKR4 ligands at a concentration representing about 3-times the EC50 value of β-arrestin2 recruitment. To determine chemokine-mediated recruitment of signaling molecules, basal NetBRET values were subtracted for each condition and the area under the curve (AUC) for the first 3 min of stimulation were calculated as depicted in Figure 3B. A comprehensive analysis revealed that CCL19, CCL21, and CCL25 selectively and efficiently recruited GRK3 to ACKR4, whereas no interaction of ACKR4 with GRK4, GRK5, or GRK6 was observed (Figure 3C). Dose-response kinetic analysis revealed similar EC50 values for the recruitment of GRK3 (Figure 3D) by CCL19 (EC50~42 nM), CCL21 (EC50~142 nM), and CCL25 (EC50~147 nM), as determined for the recruitment of β-arrestin2 by these ACKR4 agonists. Chemokine-mediated GRK3 recruitment to ACKR4 was fast, reaching its maximum within a minute (Figure 3E), and preceded the recruitment of β-arrestin1 and β-arrestin2 (Figure 3F). A less pronounced chemokine-mediated BRET signal was also observed between GRK2 and ACKR4 (Figures 3C,H). Subsequent dose-response kinetic analysis for GRK2 (Figure 3G) revealed EC50 values for CCL19 (EC50~35 nM), CCL21 (EC50~129 nM), and CCL25 (EC50~87 nM) that are comparable to those for GRK3. The chemokine-mediated interaction between GRK2/3 and ACKR4 was not as transient as one could expect, which can be explained by the spontaneous trafficking of ACKR4 that continuously deliver receptor molecules to the plasma membrane that can interact with GRK2/3 over time and upon chemokine triggering. Notably, steady-state interaction of GRK2/3 with the tailless variant ACKR4t was abrogated and no chemokine-mediated recruitment of GRK2 or GRK3 to ACKR4t was observed (Figure 3I). To investigate the role of GRK2/3 in the recruitment of β-arrestin to ACKR4, we treated cells with cpmd101, a known GRK2/3 inhibitor (31). Treating cells with cpmd101 reduced both basal interaction of ACKR4 with β-arrestin2 (Figure 3J), as well as chemokine-mediated β-arrestin2 recruitment to the receptor (Figures 3K,L).

**Figure 4:**
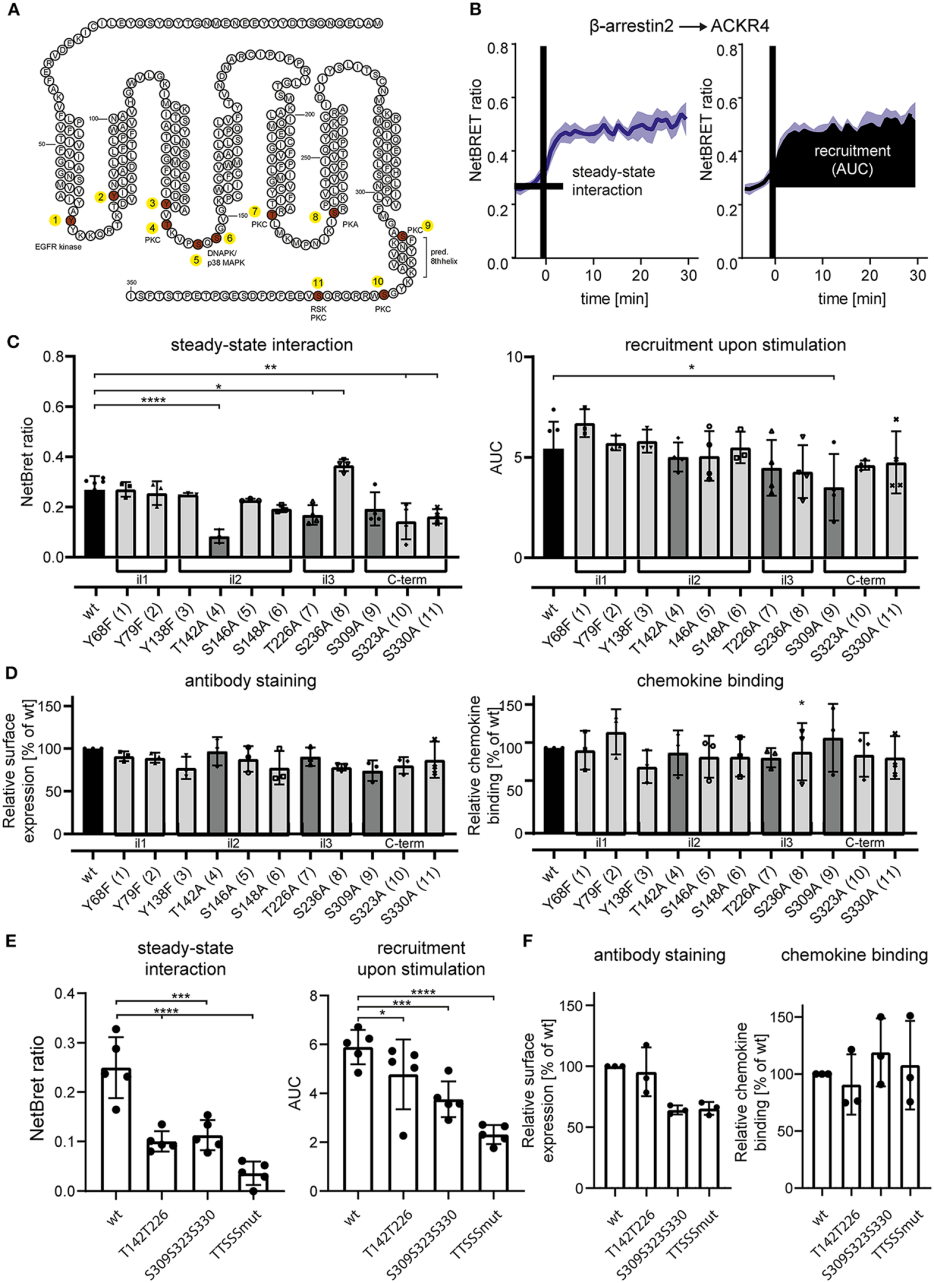
Site-specific mutation analysis of ACKR4 for putative kinase interaction sites. **(A)** Schematic representation of ACKR4. *in silico* predicted phosphorylation sites are highlighted in red and putative kinase for site-specific tyrosine or serine/threonine phosphorylation are depicted. Numbers indicate individual mutant clones analyzed in **(C)**. **(B)** HeLa cells were transfected with ACKR4-EYFP and β-arrestin2-Nluc and stimulated with 1 μg/ml (114 nM) CCL19 and steady-state interaction before chemokine stimulation and chemokine-mediated recruitment determined by NetBRET of a representative experiment out of four is shown. **(C,E)** HeLa cells were transfected with mutants of ACKR4-EYFP together with β-arrestin2-Nluc and steady-state interaction **(C)**, as well as CCL19-mediated β-arrestin2 recruitment was determined as outlined in **(B)**. **(D,F)** HeLa cells were transfected with mutants of ACKR4-EYFP and surface expression using antibody staining or chemokine binding (25 nM CCL19^Dy649P1^ at 8°C) was determined by flow cytometry. *n* = 3–5.

Notably, although cpmd101 treatment interfered with the recruitment of β-arrestin to ACKR4, the interaction was not completely abolished, suggesting that other kinase(s) contribute to potential ACKR4 phosphorylation and subsequent β-arrestin recruitment. To address this, we searched for putative serine/threonine phosphorylation sites of ACKR4. *In silico* studies using NetPhos 2.0 server (http://www.cbs.dtu.dk/services/NetPhos/) and NetPhosK 1.0 server (www.cbs.dtu.dk/services/NetPhosK) predicted several putative phosphorylation sites or protein binding motifs for ACKR4 (Figure 4A). In order to validate these putative phosphorylation and kinase binding sites of ACKR4, we performed site-directed mutagenesis to exchange tyrosine residues for phenylalanine and serine/threonine residues for alanine and determined steady-state interaction and CCL19-driven recruitment of β-arrestin2 by BRET as depicted in Figure 4B. Whereas none of the tyrosine mutants affected steady-state interaction or stimulation-dependent recruitment of β-arrestin2 to ACKR4, a number of serine/threonine single point-mutants significantly reduced the interaction between β-arrestin2 and ACKR4 (Figure 4C). Most prominently, ACKR4_T142A_ showed severely impaired steady-state interaction with β-arrestin2 without affecting the chemokine-driven β-arrestin2 recruitment. Importantly, none of these ACKR4 mutants showed significantly impaired surface expression or CCL19^Dy649P1^ binding capabilities (Figure 4D). Additional sites affecting the steady-state interaction with β-arrestin2 include ACKR4_T226A_, ACKR4_S309A_, ACKR4_S323A_, and ACKR4_S330A_, which, together with ACKR4_T142A_, are predicted as putative PKC phosphorylation sites (Figures 4A,C). Thus, we generated additional mutants, where the two threonine residues (ACKR4_T142T226_), the three serine residues (ACKR4_S309S323S330_) or the combination thereof (ACKR4_TTSSSmut_) were replaced by alanines. Notably, steady-state interaction of ACKR4_TTSSSmut_ with β-arrestin2 was profoundly reduced, whereas the other two mutants showed an intermediate phenotype (Figure 4E). Similarly, ACKR4_TTSSSmut_ showed a significantly decreased ability to recruit β-arrestin2 upon CCL19 stimulation (Figure 4E), while retaining their surface expression and chemokine binding abilities (Figure 4F).

Taken together, these data demonstrate that chemokine triggering selectively recruits GRK3, and to a lesser extent GRK2, to ACKR4 and suggest that GRK2/3 and other serine/threonine kinases contribute to the recruitment of β-arrestins to the receptor. Although, mutating selected serine and threonine residues is not a direct proof that these residues are indeed phosphorylated by GRK2/3, our data provide evidence that these residues are critical for β-arrestin recruitment.

### β-Arrestins Contribute to, but Are Dispensable for Chemokine Uptake

**Figure 5:**
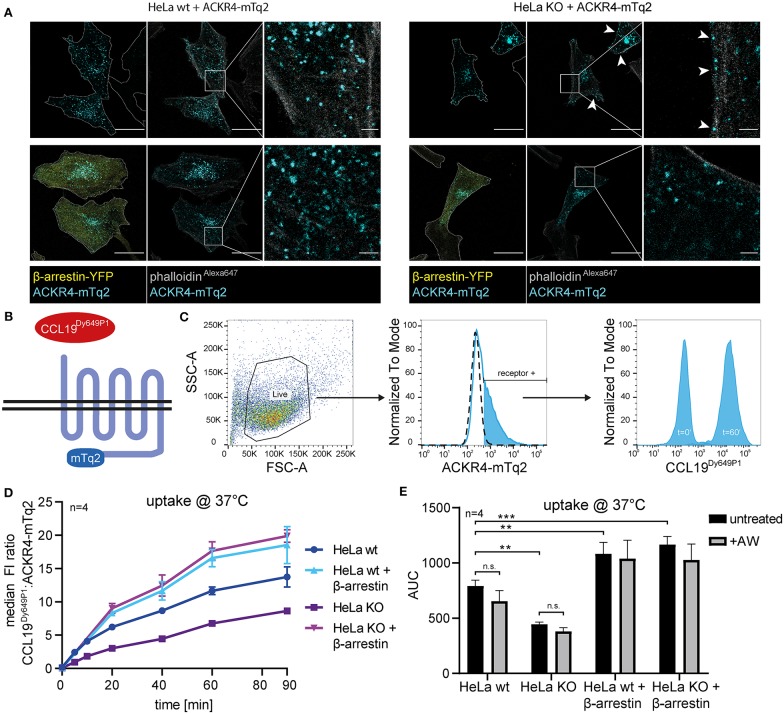
β-arrestins contribute to, but are dispensable for CCL19 uptake. **(A)** Wild-type (wt) HeLa cells or β-arrestin1 and β-arrestin2 double gene targeted HeLa cells (KO) were co-transfected with ACKR4-mTq2 and β-arrestin2-YFP or empty vector, fixed, stained with phalloidin^Alexa647^ and analyzed by confocal microscopy. Cell shapes are marked with a dashed line (outer-left panels). Scale bar = 25 μm or 2.5 μm for zoomed images. **(B)** Scheme of CCL19^Dy649P1^ binding to mTq2-tagged ACKR4. **(C)** HeLa cells (ut, dashed line) were transfected with ACKR4-mTq2 (blue line) and incubated for 0 (*t* = 0) or 60 min (*t* = 60) at 37°C with 5 nM fluorescently labeled CCL19^Dy649P1^. ACKR4-mTq2 expression and chemokine uptake (by receptor + cells) was determined by flow cytometry. One representative experiment including gating strategy is shown. **(D,E)** HeLa wt or HeLa KO cells over-expressing or not β-arrestin2 were transfected with ACKR4-mTq2 and incubated with 5 nM CCL19^Dy649P1^ for indicated times and chemokine uptake determined by flow cytometry. Mean values and SEM **(D)** or SD **(E)** are shown **(D)**. Cumulative chemokine uptake over time, as determined by the area under the curve (AUC) of the experiments shown in **(D)**. Where indicated, cells were exposed to a short acidic wash to remove surface bound, but not internalized CCL19^Dy649P1^. *n* = 4.

To assess the role of β-arrestins in steady-state trafficking and chemokine scavenging by ACKR4, we exploited wild-type HeLa (HeLa wt) and β-arrestin1/β-arrestin2-double deficient HeLa (HeLa KO) cells expressing mTurquoise2-tagged ACKR4 (ACKR4-mTq2). ACKR4-mTq2 showed the expected surface and mainly vesicular localization in HeLa wt cells (Figure 5A). By contrast, ACKR4-mTq2 predominantly associated with the plasma membrane in HeLa KO cells and was less present in vesicular structures (Figure 5A), similarly to ACKR4t-EYFP (Figure 2F). Reconstituting HeLa KO cells by reintroducing β-arrestin2-YFP, promoted the re-localization to predominantly vesicular and surface localization of ACKR4-mTq2 (Figure 5A), supporting the notion that β-arrestins control the steady-state trafficking of ACKR4. To asses ACKR4-mediated chemokine scavenging, we incubated transfected HeLa cells with fluorescently labeled CCL19^Dy649P1^ for various time points at 37°C (Figures 5B,C). CCL19^Dy649P1^ was steadily taken up over time by ACKR4 in HeLa wt cells (Figure 5D). Exposing cells to a short acidic wash hardly reduced chemokine-derived fluorescence, indicated that CCL19^Dy649P1^ was indeed internalized (Figure 5E). Remarkably, uptake of CCL19^Dy649P1^ was significantly reduced by roughly ~40–50% in HeLa KO cells, but was not completely abolished (Figures 5D,E). Moreover, overexpression of β-arrestin2 in either HeLa wt or HeLa KO cells significantly enhanced CCL19^Dy649P1^ uptake (Figures 5D,E).

In summary, we show that β-arrestins interact with ACKR4 in the steady-state and contribute to the spontaneous trafficking of the receptor. Furthermore, we demonstrate that β-arrestins enhance the scavenging activity of ACKR4, but are dispensable for chemokine uptake.

## Discussion

ACKR4 plays an important role in the regulation of immune cell migration by shaping local chemokine gradients (20, 21). The molecular mechanism how ACKR4 scavenges its cognate ligands remains poorly understood. Initially, biotinylated CCL19 was detected in vesicular structures of ACKR4 transfected MEFs derived from β-arrestin1/β-arrestin2 double-deficient mice, suggesting that chemokine uptake by ACKR4 is not critically dependent on β-arrestins (16). By contrast, CCL19 stimulation of an ACKR4 transfected osteosarcoma cell line was shown to result in the translocation of β-arrestin2-GFP to vesicular structures (26). In addition, chemokine stimulation recruited β-arrestin1 and β-arrestin2 to ACKR4 using slit-galactosidase and BRET assays in CHO cell transfectants (26). This later study is in line with the common concept of a β-arrestin-dependent receptor trafficking route taken by class A GPCRs (27). In the present study we show that CCL19, CCL21, and CCL25 effectively recruit β-arrestin1 and β-arrestin2 to engaged ACKR4, which confirms the study by Watts and colleagues (26). In addition to that study, we found that β-arrestin already interacts with ACKR4 prior to chemokine stimulation and that this steady-state interaction occurs at vesicular structures. Notably, an ACKR4 mutant lacking its C-terminus not only failed to interact with and recruit β-arrestins, it also lost its vesicular localization and showed an impaired capacity to take up chemokines. Interestingly, a C-terminally truncated variant of ACKR2 also fails to recruit β-arrestins, but was still able to scavenge chemokines (29), whereas a C-terminal deletion variant of ACKR3 (31, 53) showed a similar absence of chemokine scavenging behavior as ACKR4t. Together with the finding that overexpression of β-arrestin2 enhanced chemokine uptake, our data indicate that β-arrestins control steady-state trafficking of ACKR4 and contributes to an enhanced chemokine scavenging activity. However, we also provide experimental evidence that β-arrestins are dispensable for chemokine uptake by ACKR4, as β-arrestin1/β-arrestin2-double deficient HeLa cells are still able to internalize chemokines although less efficient that wild-type cells. Notably, CCL19 uptake by ACKR4 was shown to be partially reduced in HEK293 cells treated with methyl-β-cyclodextrin to deplete cholesterol or in cells overexpressing caveolin-1 or a dominant-negative form of dynamin, but not in cells overexpressing a dominant-negative form of Eps15 or Rab5 (16). These data conjointly suggest, that ACKR4 and likely other ACKRs utilize additional routes of endocytosis compared with canonical chemokine receptors.

Due to the lack of canonical G protein-dependent signaling, ACKRs were initially considered to be silent receptors. More recently, ACKR3 was described to execute a signaling bias toward β-arrestins leading to MAP kinase activation (24), an alternative signaling pathway for canonical class A GPCRs (27). β-arrestin signaling usually relies on GPCR kinase recruitment and subsequent receptor phosphorylation. Consistent with this concept, GRK2 (and partially GRK5) recruitment was shown to induce ACKR3 phosphorylation upon chemokine stimulation (31). Here, we identified that GRK3 and GRK2, but no other GRK, are selectively recruited to chemokine engaged ACKR4 and that GRK2/3 recruitment precedes the recruitment of β-arrestins, pointing to a remarkable specificity of GRKs for different ACKRs. Inhibiting GRK2/3 by cpmd101 partially, but significantly reduced steady-state interaction as well as chemokine-driven recruitment of β-arrestins to ACKR4. However, we did not find any experimental evidence for a β-arrestin-dependent or independent phosphorylation of Erk1/2 and Akt upon chemokine triggering of ACKR4. Our data are thus in line with a previous study on ACKR4 showing no Erk1/2 activation (26) and one on the adrenergic receptor showing that β-arrestins are dispensable for Erk1/2 phosphorylation (54).

In conclusion, it emerges that distinct GRKs are recruited to ACKRs (GRK2/5 for ACKR3; GRK2/3 for ACKR4) upon ligand stimulation, which phosphorylate C-terminal serine/threonine residues of the receptor (31) and thereby recruit β-arrestins. We herein further provide evidence that β-arrestins control steady-state trafficking of ACKR4 and promote chemokine uptake. However, it is becoming clear that β-arrestins are dispensable for chemokine scavenging by ACKR2 (29), ACKR3 (30, 31) and ACKR4 (16). The fact that GRKs are recruited to and phosphorylate the receptors strongly indicates that ACKRs are not silent receptors, but are able to elicit alternative, yet unknown signaling pathways. This is most convincingly supported by the fact that mice lacking ACKR3 die at birth with ventricular septal defects and semilunar heart valve malformation (55), while mice expressing a chemokine scavenging deficient ACKR3 are vital (31).

## Data Availability Statement

Datasets for this study are deposited on Zenodo and are publicly available under a Creative Commons Attribution 4.0 International license, doi: 10.5281/zenodo.3631895.

## Author Contributions

CM and DL designed the studies and wrote the manuscript. CM, AS, MA, and IK performed the experiments. GD'A and MU contributed HeLa KO cells. CM, AS, MA, MT, and DL analyzed the data. DL supervised the overall study.

## Conflict of Interest

The authors declare that the research was conducted in the absence of any commercial or financial relationships that could be construed as a potential conflict of interest.
