# Supplementary material for: ACKR4 Recruits GRK3 Prior to β-Arrestins but Can Scavenge Chemokines in the Absence of β-Arrestins
Source: Front Immunol. 2020 Apr 22;11:720. doi: 10.3389/fimmu.2020.00720 (PMC7188906; doi:10.3389/fimmu.2020.00720)
Supplement: Supplementary file 1 [file Data_Sheet_1.docx]

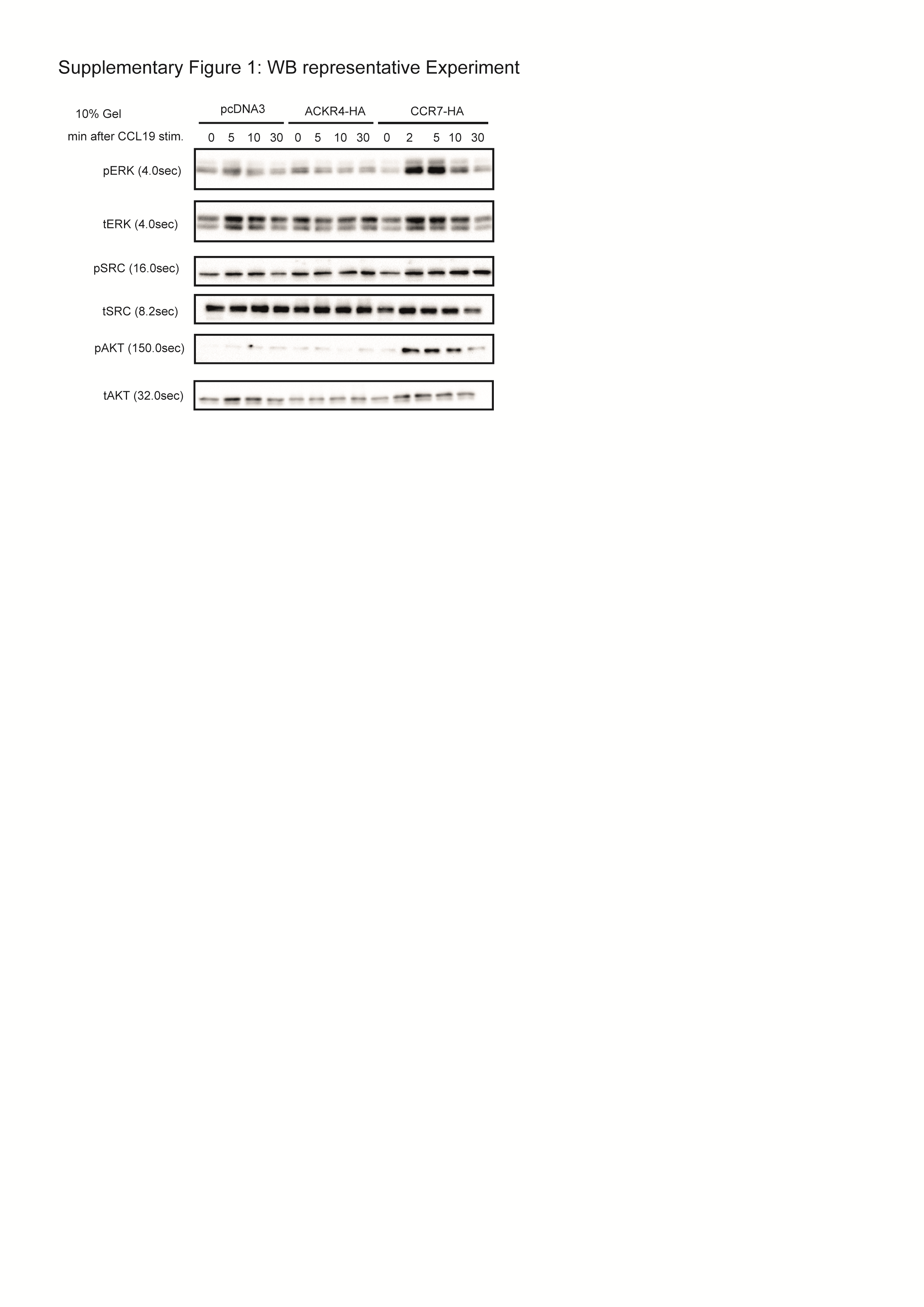


**Supplementary Figure 1:** CCL19-mediated phosphorylation of Erk1/2, Src and AKT by CCR7 but not ACKR4. Mock transfected (pcDNA3) and ACKR4-HA or CCR7-HA transfected HeLa cells were stimulated with 1µg/ml CCL19 and total versus phospho-Erk1/2, total versus phospho-Src, and total versus phospho-Akt was determined by Western blot densitometric analysis. One representative set of blots out of four is shown. Quantification of all experiments are shown in Figure 1E-G.


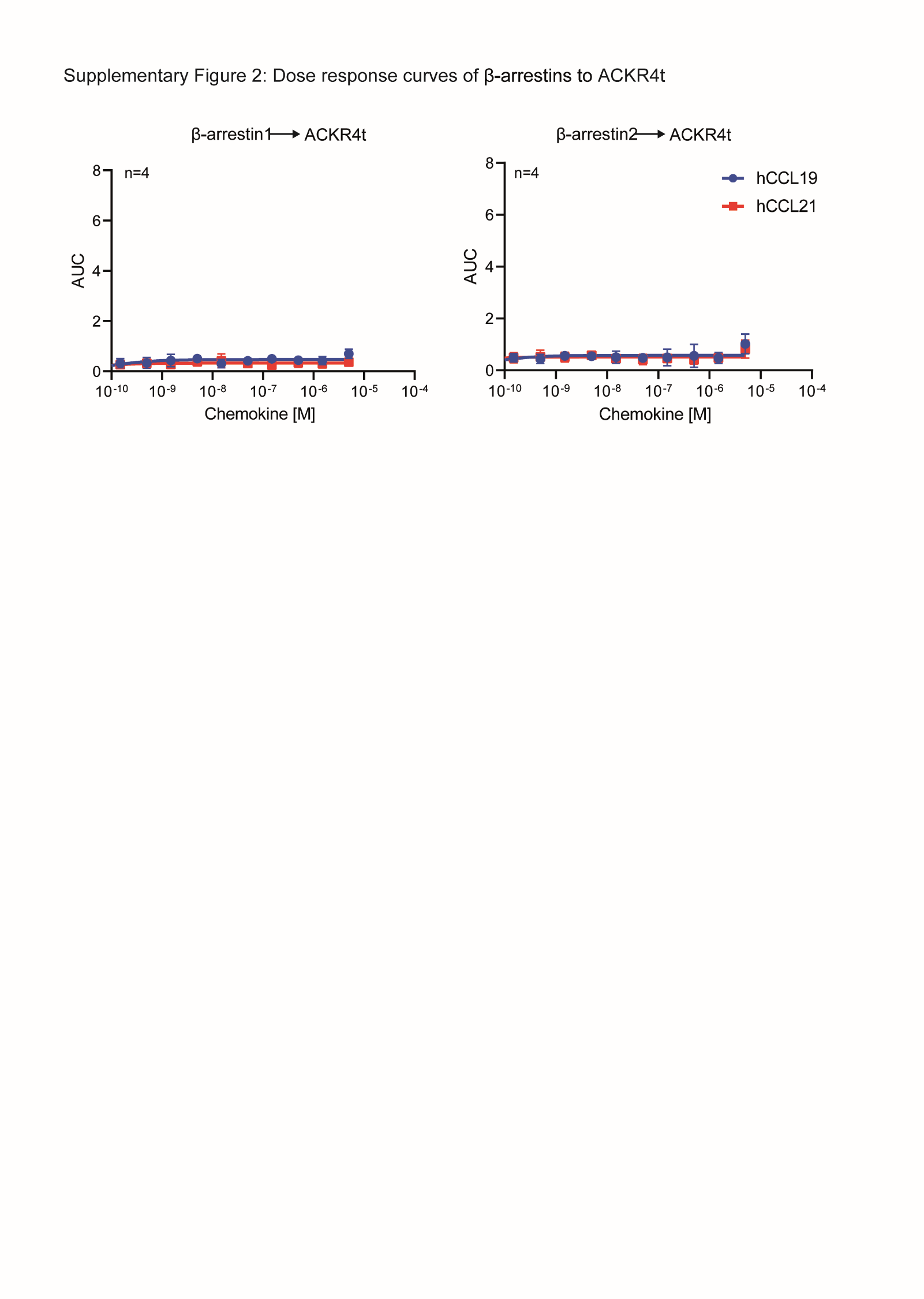


**Supplementary Figure 2:** ACKR4t does not recruit β-arrestins in a dose dependent manner. HeLa cells were transiently co-transfected with ACKR4t-EYFP and β-arrestin1-Nluc or β-arrestin2-Nluc, respectively. Area under the curve of NetBRET was determined for CCL19 or CCL21 stimulated cells over 29.5 minutes. Mean and SEM out of 4 independent experiments is shown.


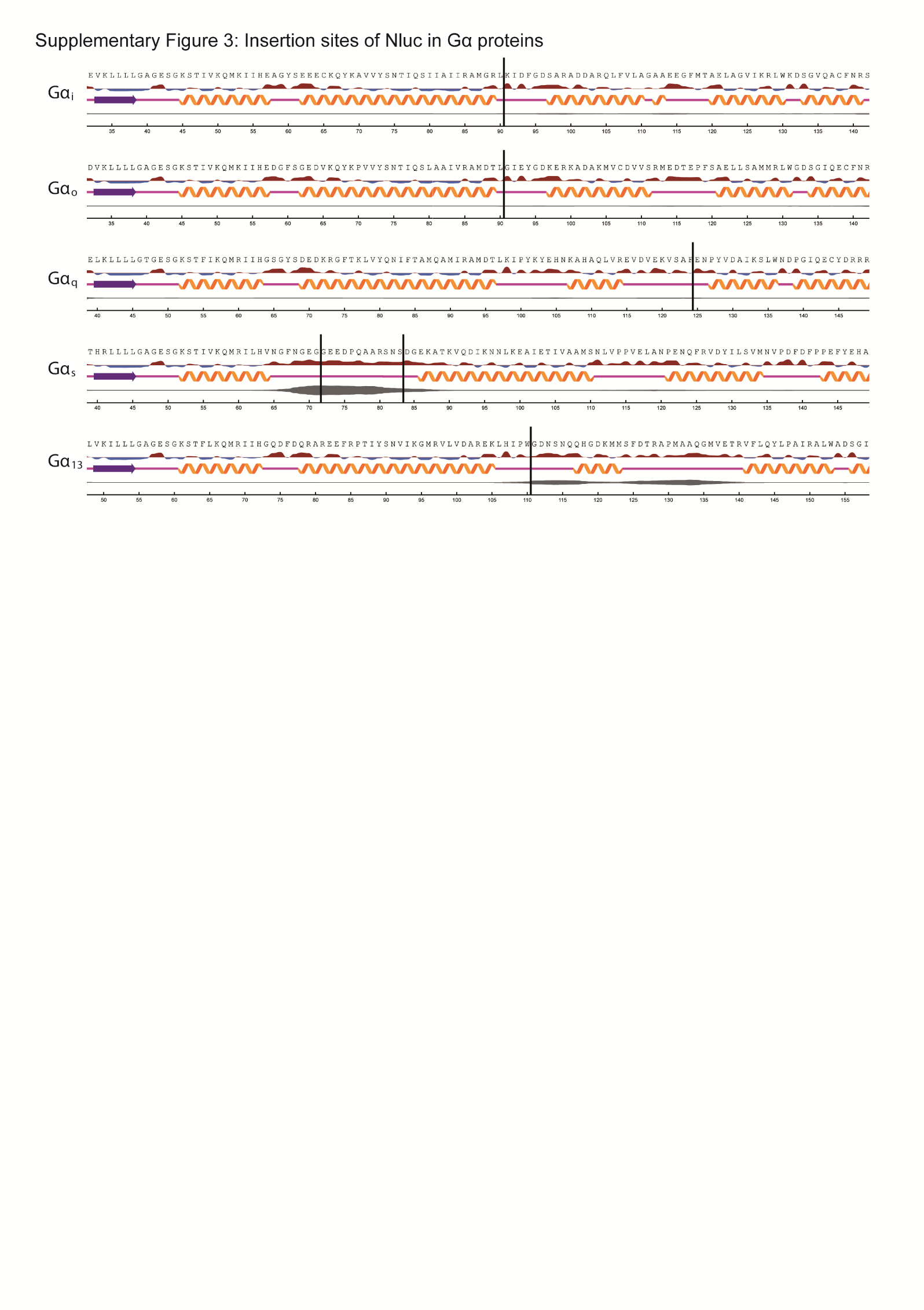
 **Supplementary Figure 3.** Schematic representation of the secondary structures of Gα subunits as predicted by NetSurfP 2.0. Sites where BRET sensors were introduced are indicated by a black vertical line. For Gα_s_, the amino acids in between the two lines have been replaced by the sensors.
